# Integrated co-expression analysis of regulatory elements (miRNA, lncRNA, and TFs) in bovine monocytes induced by *Str. uberis*

**DOI:** 10.1038/s41598-023-42067-4

**Published:** 2023-09-12

**Authors:** Somayeh Sharifi, Abbas Pakdel, Mohammad Hossein Pakdel, Raana Tabashiri, Mohammad Reza Bakhtiarizadeh, Ahmad Tahmasebi

**Affiliations:** 1 Department of Animal Science, College of Agriculture, Isfahan University of Technology, Isfahan, 84156-83111 Islamic Republic of Iran; 2 Department of Plant Molecular Biotechnology, National Institute of Genetic Engineering and Biotechnology (NIGEB), Tehran, Islamic Republic of Iran; 3 Agricultural Biotechnology Department, Faculty of Agriculture, Tarbiat Modares University, Tehran, Islamic Republic of Iran; 4 Department of Animal and Poultry Science, College of Aburaihan, University of Tehran, Tehran, 3391653755 Islamic Republic of Iran; 5 Institute of Biotechnology, Shiraz University, Shiraz, 71946-84334 Islamic Republic of Iran

**Keywords:** Biotechnology, Computational biology and bioinformatics, Molecular biology, Systems biology, Diseases

## Abstract

Non-coding RNAs, including long non-coding RNAs (lncRNAs) and microRNAs (miRNAs), together with transcription factors, are critical pre-, co-, and post-transcriptional regulators. In addition to their criteria as ideal biomarkers, they have great potential in disease prognosis, diagnosis, and treatment of complex diseases. Investigation of regulatory mechanisms in the context of bovine mastitis, as most common and economic disease in the dairy industry, to identify elements influencing the expression of candidate genes as key regulators of the mammary immune response is not yet fully understood. Transcriptome profiles (50 RNA-Seq and 50 miRNA-Seq samples) of bovine monocytes induced by *Str. uberis* were used for co-expression module detection and preservation analysis using the weighted gene co-expression network analysis (WGCNA) approach. Assigned mi-, lnc-, and m-modules used to construct the integrated regulatory networks and miRNA-lncRNA-mRNA regulatory sub-networks. Remarkably, we have identified 18 miRNAs, five lncRNAs, and seven TFs as key regulators of *str. uberis*-induced mastitis. Most of the genes introduced here, mainly involved in immune response, inflammation, and apoptosis, were new to mastitis. These findings may help to further elucidate the underlying mechanisms of bovine mastitis, and the discovered genes may serve as signatures for early diagnosis and treatment of the disease.

## Introduction

Bovine mastitis is defined as inflammation of the mammary gland caused by a variety of infectious agents, including bacteria, mycoplasma, yeasts, and algae^1^. Mastitis as an endemic disease, leading to the development of subclinical/chronic with 25–65% incidence worldwide or clinical (∼5% incidence worldwide) infections^2^, is implicitly associated with a reduction in the quantity and quality of milk production, loss of reproductive efficiency, and increased susceptibility of animals to other diseases^3^.

The immune response to pathogens in the mammary gland is highly complex and involves resident, recruited, and inducible immune factors^4^. Despite the extensive interaction of innate (non-specific) and adaptive (specific) immunity factors to provide adequate protection, the magnitude, duration, and efficacy of mammary gland immunity against mastitis is significantly influenced by specific aetiological agents^5^. Streptococcus uberis (*Str. uberis*), a gram-positive bacterial pathogen, is an amazingly versatile mastitis pathogen that can affect multiparous cows as well as heifers in all lactation status (milking and dry periods), with clinical or subclinical symptoms that can even persistent colonization without elevation in the somatic cell count^1,6^*.* The epidemiology of the *Str. uberis* pathogen is not fully understood. The persistence of *Str. uberis* in the infected bovine udder, due to its biochemical capabilities and ability to invade mammary cells, as well as its ability to form biofilm and capsule, promotes the development of chronic mammary infections and allows the pathogen to change from an environmental to a cow-associated form^7,8^.

More recently, analysis of the transcriptome profile of the bovine mammary epithelial cells in response to infection revealed several genes (e.g., TNF, IL6, IL8, IL10, TP53, TGFB1), gene lists (e.g., IL-10 and IL-6 signaling), and miRNAs (e.g., mir-155, mir-204)^9^, which are responsible for a wide range of inflammatory and immunological responses^10–12^.

Furthermore, the role of key genes in the molecular mechanisms of resistance to bovine mastitis^13^ and also in predicting drug candidates for the control and management of mastitis has been previously investigated^14^, but the regulatory elements, such as miRNAs, lncRNAs, and TFs, involved in the expression of these genes are not yet fully understood. A growing number of reports suggest a significant utility of miRNAs^15^, lncRNAs^16,17^, and TFs^16^ as biological markers of pathogenic conditions, modulators of drug resistance, and/or drug development for medical intervention.

Non-coding RNAs, untranslated RNA molecules that play a regulatory role in gene expression, are involved in many biological processes. These functional RNA molecules are divided into several groups such as small interfering RNA (siRNA), small nucleolar RNA (snoRNA), circular RNAs (circRNAs), PIWI-interacting RNA (piRNA), microRNA (miRNA), and long non-coding RNA (lncRNA)^18^. Among these, miRNAs and lncRNAs have attracted the attention of researchers in the field of immune-related gene expression signatures^19^.

MiRNAs are short non-coding RNAs of ~ 22 nucleotides in length that bind to the coding region of an mRNA, the 3′ and 5′ untranslated region (UTR), repress the translation of mRNA into protein (post-transcription) and control biological processes in humans, plants and animals^20–22^. Research has shown that when cells receive exogenous or endogenous signals, the expression of miRNAs inside the cells changes, and during the onset of disease symptoms, the expression levels of some miRNAs change to control the development of the disease^23–25^. Therefore, miRNAs can be used as early diagnostic biomarkers at the onset or progression of disease^25,26^. It has been shown that miRNAs of *Str. uberis* infection are key amplifiers of the inflammatory response networks of monocytes^27^.

LncRNAs with more than 200 nucleotides (nt)^28,29^, could not encode proteins and harbored a 5′ cap and 3′ poly (A)^30,31^. LncRNAs play roles in the level of gene transcription, epigenetic and post-transcriptional modification, and regulate the level of the immune and inflammatory response during the disease process^28,29^. Genomic imprinting, DNA methylation, splicing, and chromatin modification are some of the other roles of lncRNAs^32,33^. The inflammatory response may be promoted by the effect of lncRNAs on the transcription of certain genes^34^. Previous research has suggested that lncRNAs have the potential to become important diagnostic markers for mastitis and can be used to control and prevent this disease^35^. Recently, computational analyses showed that miRNA, lncRNA, and TFs could regulate the host immune response to bovine mastitis^19^.

This is the first report investigating regulatory elements including miRNA, lncRNA and TFs that influence the expression of candidate genes in the mammary immune response in the context of Str. uberis bovine mastitis. We used mRNA and miRNA sequencing datasets to construct an integrated co-expression network. Given the relatively low activity of some highly expressed microRNAs which in some cases correlated with a high target-to-microRNA ratio or increased nuclear localization of the microRNA, analysis based on module detection is more appropriate than making decisions based on differential expression results.

In order to explore the key regulatory elements involved in the onset and development of mastitis, several processes including module discovery, preservation analysis, module assignment, functional enrichment analysis, regulatory network construction, and network integration were used to extract miRNA-lncRNA-mRNA sub-networks and identify hub genes. In addition, target prediction for mi and lncRNAs was used to confirm the elements in the assigned modules at the sequence level.

## Material and methods

### Data sources and pre-processing

Raw RNA and miRNA sequencing data were obtained from NCBI's Gene Expression Omnibus (GEO) data repository under the accession number GSE51856 and GSE51858 respectively. These data contained transcriptome profiles of milk samples related to five Holstein Friesians cows infected with *Str. uberis 0140* colonies, as well as five control animals, inoculated with saline only, in the middle of first lactation at the time points 0, 12, 24, 36, and 48. In brief, milk-derived CD14^+^ monocytes with more than 95% purity were isolated by fluorescence-activated cell sorting (FACS). Total RNA was extracted using mirVana RNA Isolation Kit (Ambion, Austin, TX) and MicroRNA was extracted using mirPremier microRNA Isolation Kits (Sigma-Aldrich, Steinheim, Germany) from FACS-isolated cell populations. The Illumina HiSequation 2000 was used for sequencing (50-bp single-end) with three or four samples and seven or eight samples multiplexed per lane for mRNAs and miRNAs respectively. Finally, 50 RNA-Seq libraries (25 Infected and 25 control samples) containing 50-bp single-end reads and 50 miRNA-Seq libraries (25 Infected and 25 control samples) containing 50-bp single-end reads were generated. In the original paper, more details on preparing data can be found^21^.

FastQC (version 0.11.9) was used for quality check of raw RNA-Seq and miRNASeq reads^36^. These data did not have any adaptor sequence. Bases/reads for both RNA and miRNA reads with Low-quality were removed by Trimmomatic (version 0.32)^37^. The trimming criteria for RNA-Seq data were MAXINFO: 40:0.9 MINLEN:36 TRAILING:3 and for miRNA-Seq data was MAXINFO:18:0.9 MINLEN:18 TRAILING:3.

MAXINFO trimmer, performs an adaptive quality trim, balancing the benefits of retaining longer reads against the costs of retaining bases with errors. TRAILING, Remove trailing N bases, if below quality 3. In RNAseq studies, when the length of the data is less than a normal limit, they should be removed because they will cause errors in other steps such as multiple alignments, so we used the MINLEN option to remove reads that fall below the specified minimal length. The quality of reads after trimming was monitored again by FastQC.

Hisat2 (version 2.2.1) was used for the alignment of clean reads of RNA data to index bovine reference genome using disable spliced alignment option. Indexing of genome constituted by hisat2-build function (Bos_taurus. ARS-UCD1.2 version from ENSEMBL database**,** released Apr2018)^38^. Then, the mapped RNA reads annotated by the Ensembl bovine GTF file (GCA_002263795.2 genome, release 103) were counted using HTSeq-count (version 2.0.1)^39^.

For miRNA data, Bowtie software (version 1.2.2)^40^ with “-n 1 -e 80 –best –strata -a” options were used to align reads to hairpin bovine miRNA sequences obtained from the miRBase database (v22.1)^41^. Defined alignment options report only those miRNA reads in the best alignment “stratum,” with no more than one mismatch. Reads that did not uniquely align with the miRBase were discarded.

Then, quantify the expression of each miRNA and the expression matrix was generated by SAMtools (version 1.7)^42^.

Raw counts of RNA were processed by the voom function in the limma package (R software version 4.1.0) to convert them into log2 counts per million (logCPM) and provide mean–variance relationship plot^43^. The calcNormFactors function implemented in edgeR was used for obtaining the TMM normalization factors^44^.

To gather more reliability of the genes, genes in both expression matrices with expression levels ≥ 1 CPM in at least three samples followed by the standard deviation larger than 0.25 were kept for further analysis^45^. lncRNAs in the mRNA matrix were identified and separated using the Ensembl bovine GTF file (GCA_002263795.2 genome, release 103). To identify bovine transcription factors (TF)s in the mRNA matrix, the AnimalTFDB3.0 database was used^46^. Finally, three matrices including miRNA, lncRNA, and mRNA (including TFs) were used for module detection separately.

### Module construction based on the WGCNA algorithm

A systems biology approach using WGCNA^47^ was applied to explore the complex relationships between genes and phenotypes by constructing scale-free co-expression networks. In this research, 3 weighted co-expression networks for healthy samples of mi, lnc, and mRNA expression datasets were constructed separately. Since coexpression analysis is very sensitive to outliers, the outlier samples were detected and excluded from the original matrices. Detection of outliers was performed by calculation of adjacency matrices of expression matrices. The sample network connectivity according to the distances was standardized and samples with standardized connectivity less than − 2.5 were detected as outliers. Then goodSamplesGenes function in the WGCNA package was applied to determine whether the sample data were complete as well as exclude the unqualified genes. The construction processes of gene co-expression networks were the same in the three construction networks except for a number of parameters that are mentioned following. First, the optimal value of soft threshold power β, which is a weighted parameter of the adjacent function, was obtained by the pickSoftThreshold. Then, appropriate soft-thresholding power was calculated to be confident of the scale-free topology of the constructed network^48^. Second, the blockwiseModules function in the WGCNA package used for module detection, with the major parameters: power = 20, corType = “bicor”, networkType = “signed”, TOMType = “signed”, maxBlockSize = 17,000, minModuleSize = 30, reassignThreshold = 0, mergeCutHeight = 0.25 for mRNA. The power for miRNA and lncRNA changed to 12 and 10 respectively. According to the smaller size of the miRNAs and lncRNAs in comparison to the mRNA, the minModuleSize was set to 5 for them. Noticeably, to identify the modules in three datasets, first, weighted adjacency matrices were constructed based on the soft-thresholding power, and were transformed into the topological matrix (TOM). The TOM, which describes the association strength between the genes, was used to convert the adjacency value into a TOM matrix. Then, TOM-based matrices clustered the genes into the hierarchy to get the system clustering tree by using average linkage hierarchical clustering analysis through a dynamic hybrid tree-cutting algorithm^49^. Hierarchical clustering is a widely used method for detecting clusters with a small degree of dissimilarity for co-expressed elements. The dissimilarity measure based on TOM can be used as input in hierarchical clustering, then modules were defined as branches of a cluster tree, and each module was labeled by a unique color using the static tree cutting method. Eventually, mi, lnc, and mRNAs with similar expression profiles were divided into modules named mi-, lnc-, and m-modules, respectively. Third, the correlations between the module eigengenes were used to detect the highly similar modules to further merge.

### Preservation analysis

Preservation status (preserved, semi-preserved, and non-preserved) of connectivity patterns of the genes in the detected m-modules of healthy samples in comparison with infected samples were detected by modulePreservation function in the WGCNA package. Two composite module preservation statics, Zsummary, and medianRank, were applied to evaluate the preservation status detected based on connectivity and density of genes included in each module^50^. The Zsummary < 5 was considered as the threshold for non-preserved modules, between 5 and 10 was considered as the semi-preserved modules, and greater than 10 evidence was considered as the preserved modules. To evaluate the statistical significance of both module preservation statics, the permutation method (N = 200 permutations) was used.

### Assign the relationship between modules

Eigengene (MEs) of elements were used to assign the mi- and lnc-modules to non- or semi-conserved m-modules and also mi-modules with lnc-modules. Pearson correlations and associated p-values were estimated between the module MEs of given modules using corAndPvalue function in the WGCNA package. The MEs, considered representative of the expression profiles in a module, refer to the first principal component of a given module. Negative or positive correlations greater or less than + 0.70 and -0.7 with a significant statistic (adjusted *P value* < 0.05) respectively, were defined as regulators of interest modules. Negative correlations suggest that mi-modules might inversely regulate m-modules. Hence, mi-modules only with a significantly negative correlation (adjusted *P value* < 0.05) were considered regulatory relations between mi- and m-modules.

### Target prediction for miRNAs and lncRNAs

To make a stronger biological connection between mi- and lnc-modules with assigned modules as upstream regulatory elements, the target prediction was performed for miRNAs and lncRNAs. Therefore, genes in assigned modules were confirmed by prediction software considered as targets.

To avoid false positive results, lncRNAs or mRNAs assigned to miRNAs that are confirmed by the predicted targets of at least two out of the three applied software including miRanda (version 1.9)^51^, RNAhybrid (version 2.2.1)^52^, and RNA22 (version 2)^53^ considered as targets of given miRNAs. The minimum free energy threshold was set as –15, and other parameters were set as default settings for all used software. In this respect, bovine mature miRNA sequences were obtained from the miRBase database (version 22.1), and bovine 3′ and 5′ UTR sequences and coding sequence (CDS) of mRNAs were retrieved from the BioMart database (https://www.ensembl.org/biomart). The sequences of lncRNAs were obtained by using the gget tool (version 0.2.6) which is a UNIX base tool for downloading sequences by using their Ensemble IDs.

LncTar tool (version 1.0)^54^ was used to predict the targets of lncRNAs assigned to CDS regions of mRNAs.

### Hub mRNAs verification

It is known that all presented genes in a module were not important to be linked with the subject of interest. For more focus on the most important mRNAs in each module, mRNAs with a central role in the co-expression network with high connectivity within each module, as hub genes used for enrichment analysis. The MEs value in each module and signedKME functions of the WGCNA package were used to identify the hub elements in the detection modules.

For each mRNA, eigengene-based connectivity (k_ME_) and a "fuzzy" measure of module membership were determined by the correlation between its expression profile with the MEs of a given module. The k_ME_ measure is highly related to intramodular connectivity (k_ME_). Highly connected intramodular hub elements tend to have high module membership values to the respective module. Hubs in modules were determined as the elements with k_ME_ ≥ 0.8 in mastitis samples and k_ME_ ≤ 0.8 in healthy samples^47^.

### Functional enrichment analysis

To assess the putative functions associated with the genes in each m-modules based on preservation status, and detected hub genes, GO terms were performed using the EnrichR online analysis tool (https://maayanlab.cloud/Enrichr/)^55^. Only significant terms with adjusted *p-values* < 0.05 were considered.

### Construction of the regulatory network

In order to identify and visualize the most important regulatory elements and pathways in assigned modules, the following steps were performed:

1. All the interactions related to mi, and lnc-modules and their verified targets were used as inputted data to Cytoscape software (version 3.9.1)^56^.
2. Maximum click centrality (MCC) topological analysis methods of CytoHubba application^57^ in Cytoscape were used to screen the five highly connected genes in each module-module interaction network separately.
3. Integrated network constructed to illustrate regulatory pathways, upstream main regulators, and miRNA–lncRNA–mRNA interaction sub-networks.

## Results

### RNA-Seq and miRNA-Seq preprocessing

**Figure 1:**
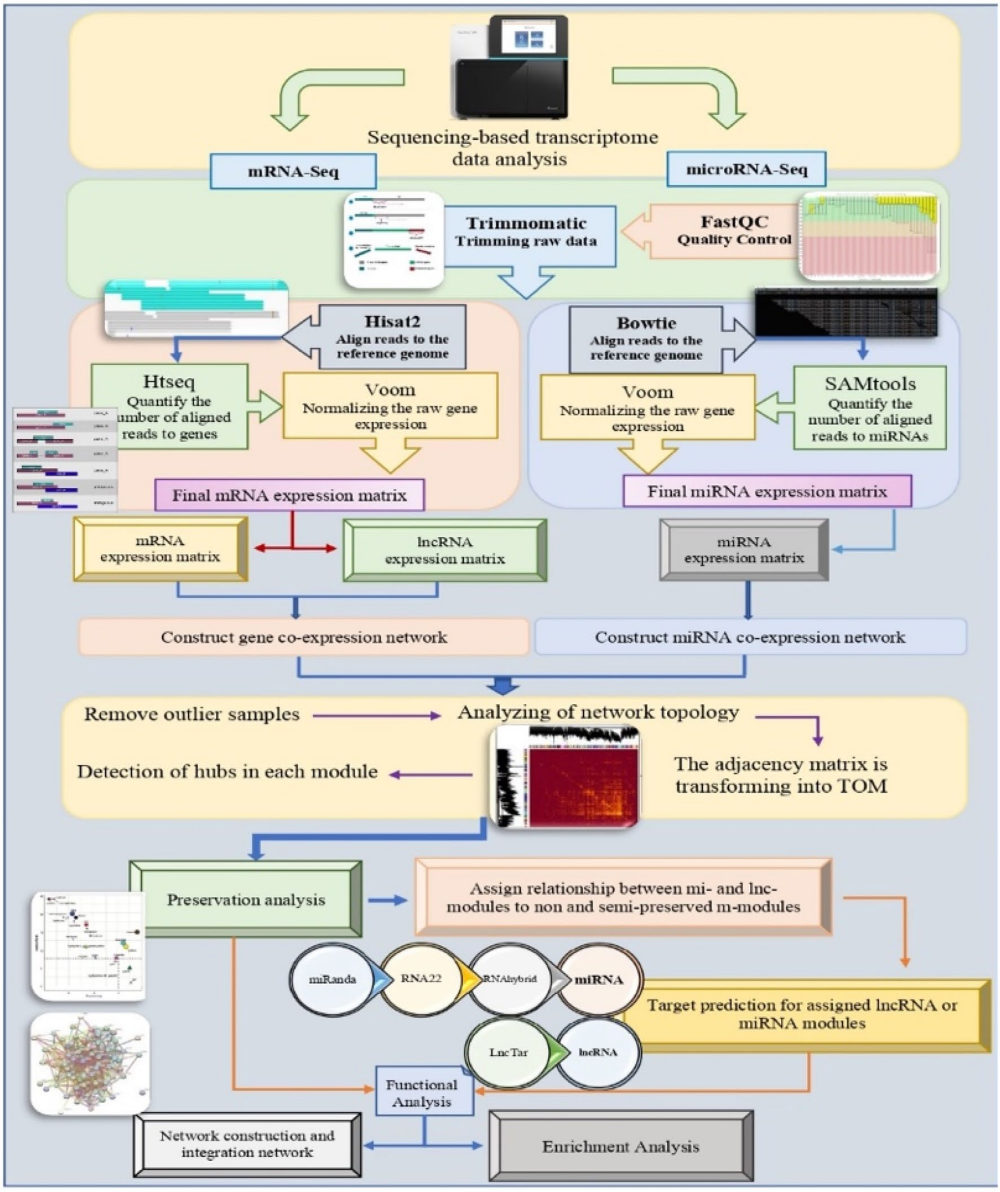
The workflow of this study.

The overall analysis workflow is shown in Fig. 1. After preprocessing, from 1,876,238,611 raw reads belonging to 50 RNA-seq samples, 1,601,194,408 clean reads remained with an average of 32,023,888 per sample. After alignment with an average of 84.86% to the bovine reference genome, counting, and integration of samples, two expression matrices containing 26,127 mRNAs (including 767 TFs), and 1480 lncRNAs obtained.

In miRNA samples (50 miRNA-seq samples), from 702,106,058 raw reads, 650,547,536 clean reads with an average of 13,010,950 reads per sample obtained. 43.03% of the clean reads are aligned to the bovine hairpin miRNAs. To ensure no genomic contamination, mapping to the bovine reference genome was performed for some miRNA samples that have a low mapping rate to bovine hairpin miRNAs (more than 80% of reads mapped to the bovine reference genome). Mapped reads counts and integrated to achieve a miRNA expression matrix (with 997 miRNAs). The summary tables of the preprocessing and mapping status of mRNA and miRNA samples are provided in separate sheets in Supplementary Table S1 online.

Matrices achieved from primary preprocessing lead to further filtration to remove non-expressed genes based on the expression values across the majority of samples and non-informative genes based on the variation of expression between samples. Three expression matrices including 699, 1024, and 11,497, mi, lnc, and mRNAs respectively, passed all the aforementioned filters and were used for coexpression analysis**. **These three matrices are prepared in separate sheets available in Supplementary Table S2 online online. The mean–variance trend shows how the coefficient of variation of the counts depends on the count size in each database provided in Supplementary Fig. S1 online.

### Construction of weighted co-expression network

In this study, a network-based approach was applied to better understand the molecular mechanisms and regulatory elements in mastitis with *Str. uberis* infection. Three samples were defined as an outlier and were excluded for further analysis (TC.1.003 in mRNA and TC.2.001 and TC.2.003 in miRNA samples).

Genes are clustered into modules based on their co-expression by WGCNA. After the determination of the appropriate soft threshold power beta (Supplementary Fig. S1 online), 31 m-modules, 30 mi-modules, and three lnc-modules were detected by hierarchical clustering and dynamic branch cutting (Supplementary Fig. S1 online). The average number of genes per module was 360 in the m-modules (ranged from 44 in pale turquoise to 2127 in turquoise modules), 23 in mi-modules (ranged from 5 in steel blue to 54 in turquoise), and in lnc-modules size of modules were including 14 in brown, 15 in blue, and 586 in turquoise. Additionally, 728, 29, and 408 genes were reported as grey modules in m-, mi-, and lnc-modules, respectively, which contained some genes that were not assigned to any module (Supplementary Table S3 online).

### Preservation analysis

**Figure 2:**
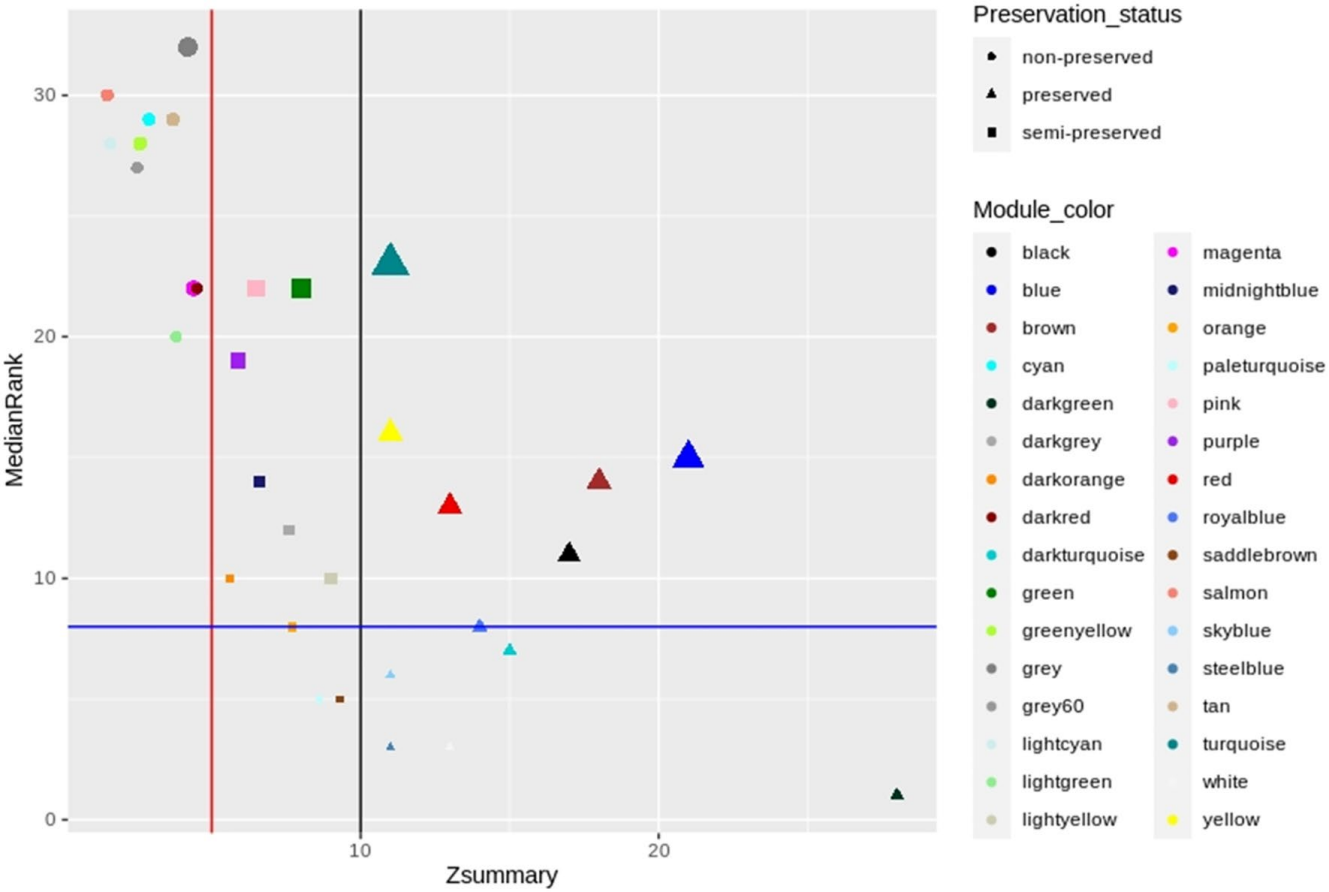
Preservation analysis based on the Zsummary criteria. The medianRank (y-axis) and Zsummary (x-axis) statistics of the module preservation. The red and black vertical lines indicate the thresholds Zsummary = 8 and Zsummary = 10 respectively. The modules with Zsummary < 5 were considered as non-preserved, Zsummary between 5 and 10 as semi-preserved and Zsummary > 10 as preserved. MedianRank of the modules close to zero indicates a high degree of preservation.

The preservation analysis of m-modules was performed to investigate the connectivity patterns between the two healthy and mastitis conditions. Connectivity patterns or network properties in the non- and semi-preserved modules altered under mastitis compared to healthy conditions, so they could represent a set of genes affected or influenced by the disease. Of 31 m-modules, 12, 9, and 10 modules were detected as preserved, semi-, and non-preserved modules respectively (Fig. 2 and Supplementary Table S4 online).

### Assigning the mi- and lnc-modules to non- and semi-preserved modules as well as mi- to lnc-modules

To explore the potential molecular mechanisms and regulatory elements responsible for mastitis disease, MEs of mi- and lnc-modules were observed to be correlated together with MEs of non- and semi-preserved m-modules. Pearson correlation (adjusted *P value* < 0.05) was used for the illustration of module relations. The assigned modules are shown in Table 1 and more details are prepared in Supplementary Tables S6, S7, and S8 online. A heatmap was drawn that provides a clear picture of the interactions between mi-, lnc-, and m-modules (Supplementary Fig. S1 online file S10).

**Table 1:** Summary of relation assigned between modules based on Pearson correlation with adjusted *P value* < 0.05.

(A) Assigned mi- to lnc-modules			
Module names	Turquoise	Blue	Brown
Blue	−0.7148		
Cyan			−0.7157
Steelblue		0.7372	0.7273

(B) Assigned mi- to m-modules			
Module names	Saddlebrown	Green	Salmon
Lightgreen	−0.7191		
Royalblue		−0.8397	
Darkgreen			−0.7433

(C) Assigned lnc- to m-modules			
Module names	Tan	Cyan	Midnightblue
Turquoise	0.76267	−0.706	0.7903

### Confirmation of assigned regulatory elements (mi and lncRNAs)’s targets by target prediction tools

The summary of the targets of mi-modules assigned to m-modules and lnc-modules confirmed by miRanda, RNA22, and RNAhybrid software, as well as common confirmation (were detected by at least two out of the three applied software) were presented in Tables 2 and 3 respectively. More details were provided in Supplementary Tables S9 and S10 online. Summary information about the target prediction of lnc-modules assigned to m-modules was presented in Table 4 and is available with more details in Supplementary Table S10 online.

**Table 2:** Summary of target prediction of mi-modules assigned to semi- and non-preserved m-modules by different prediction tools.

Software	Mi-module (number of miRNAs)	m-module (number of genes)	Number of the unique predicted targets
MiRanda	Lightgreen (18)	Saddlebrown (47)	38 mRNA, 9 TF
	Royalblue (16)	Green (652)	376 mRNA, 15 TF
	Darkgreen (15)	Salmon (211)	162 mRNA, 36 TF
RNA22	Lightgreen (18)	Saddlebrown (47)	32 mRNA, 9 TF
	Royalblue (16)	Green (652)	364 mRNA, 48 TF
	Darkgreen (15)	Salmon (211)	148 mRNA, 32 TF
RNAhybrid	Lightgreen (18)	Saddlebrown (47)	16 mRNA, 3 TF
	Royalblue (16)	Green (652)	269 mRNA, 19 TF
	Darkgreen (15)	Salmon (211)	111 mRNA, 8 TF
Common results (detected by at least two out of the applied software)	Lightgreen (18)	Saddlebrown (47)	38 mRNA, 9 TF
	Royalblue (16)	Green (652)	279 mRNA, 22 TF
	Darkgreen (15)	Salmon (211)	132 mRNA, 11 TF

**Table 3:** Summary of target prediction of mi-modules assigned to lnc-modules by different prediction tools.

Software	Mi-module (number of miRNAs)	Lnc-module (number of lncRNAs)	Number of the unique predicted targets
miRanda	Blue (46)	Turquoise (586)	508 lnc
	Cyan (22)	Brown (14)	7 lnc
	Steelblue (5)	Brown (14)	4 lnc
	Steelblue (5)	Blue (15)	1 lnc
RNA22	Blue (46)	Turquoise (586)	480 lnc
	Cyan (22)	Brown (14)	4 lnc
	Steelblue (5)	Brown (14)	4 lnc
	Steelblue (5)	Blue (15)	3 lnc
RNAhybrid	Blue (46)	Turquoise (586)	245 lnc
	Cyan (22)	Brown (14)	4 lnc
	Steelblue (5)	Brown (14)	4 lnc
	Steelblue (5)	Blue (15)	4 lnc
Common results (detected by at least two out of the applied software)	Blue (46)	Turquoise (586)	275 lnc
	Cyan (22)	Brown (14)	2 lnc
	Steelblue (5)	Brown (14)	2 lnc
	Steelblue (5)	Blue (15)	0 lnc

**Table 4:** Summary of target prediction of lnc-modules assigned to semi- or non-preserved m-modules using LncTar tool.

Software	lnc-module (number of lncRNAs)	m-module (number of genes)	Number of the unique predicted targets
LncTar	Turquoise (586)	Tan (270)	252
		Cyan (209)	199
		Midnightblue (192)	190

### Genes detected as hubs in m-modules

Between 10,769 genes clustered in m-modules (without considering grey modules), 532 genes were determined as intramodular hub elements (107 semi-, 74 non-, and 351 preserved).

The complete list of the hub genes in each m-module was presented in Supplementary Table S11 online.

### Functional enrichment analysis

To assess the putative functions associated with the modules, all the identified semi- and non-preserved m-modules and their hubs were subjected to functional enrichment analysis, separately. Functional analysis of 9 non-preserved m-modules and 10 semi-preserved modules presented in Supplementary Tables S12 and S13 respectively. Hub genes of non-preserved modules enriched biological terms mainly including negative regulation of TORC1 signaling, positive regulation of macroautophagy, positive regulation of leukocyte mediated immunity, regulation of dendritic cell cytokine production, positive regulation of I-kappaB phosphorylation, interleukins production, leukocyte differentiation, and dendritic cell cytokine production, p38MAPK cascade, toll-like receptor 2 signaling pathway, T cell activation, negative regulation of phospholipase activity, myeloid leukocyte mediated immunity, and fatty acid oxidation, positive regulation of immune cell apoptotic and negative regulation of apoptotic signaling pathways (Supplementary Table S14 online). Hub genes in the semi-preserved module generally enriched terms related to immune response including B cell, T cell activation, lymphocyte-mediated immunity, adaptive immune response, negative regulation of intrinsic and extrinsic apoptotic signaling pathways, positive regulation of cell–cell adhesion, and regulation of macrophage cytokine production (Supplementary Table S15 online).

### Regulatory networks constructed by WGCNA results

**Figure 3:**
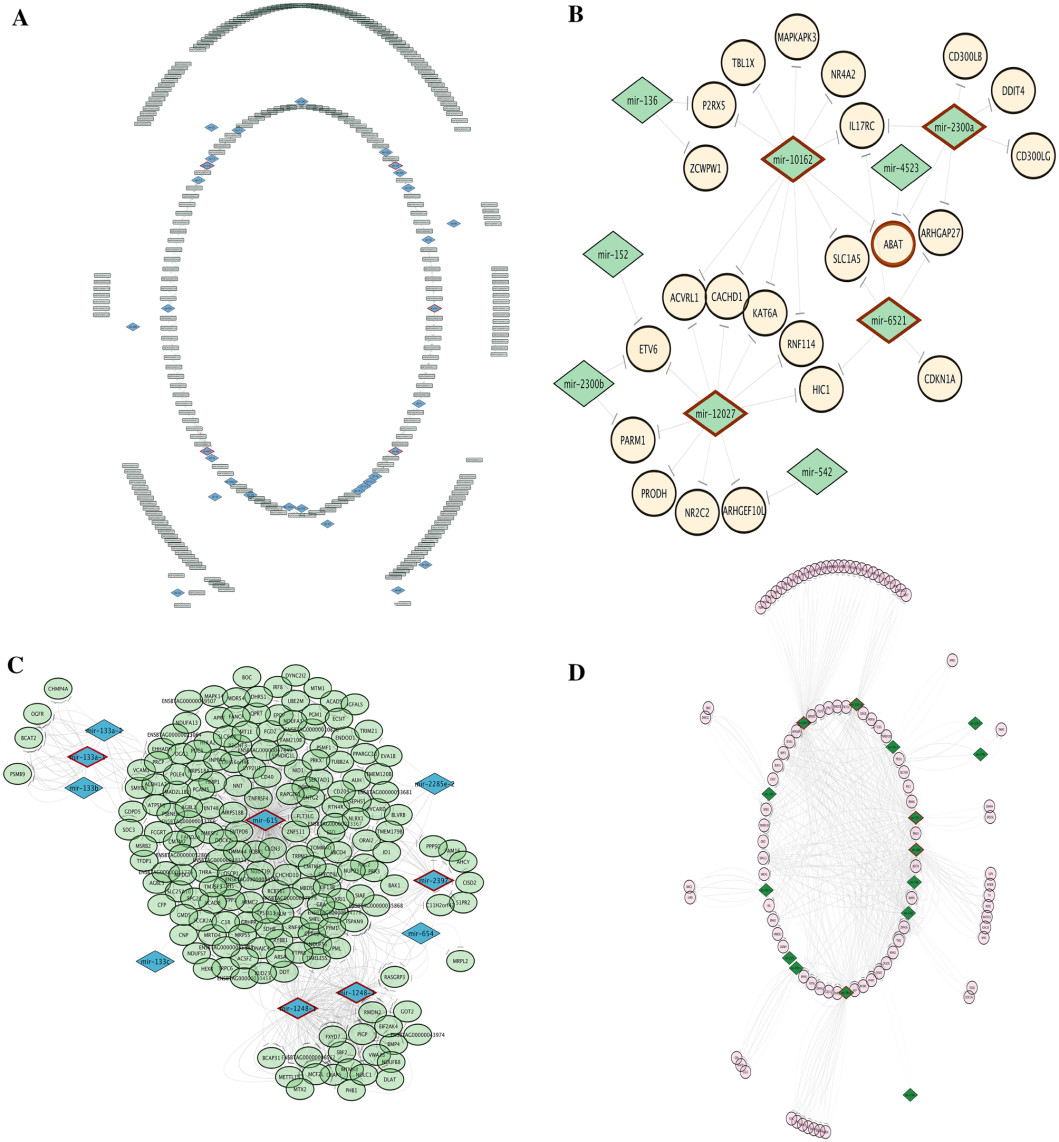
Regulatory networks of (**A**) negative relationship between blue mi-module and turquoise lnc-module, **B**) negative relationship between lightgreen mi-module and saddlebrown m-module, (**C**) negative relationship between royalblue mi-module and green m-module, (**D**) negative relationship between darkgreen mi-module and salmon m-module. miRNAs are represented with diamonds, lncRNAs with rectangles, and in m-modules ellipses show regular proteins and parallelograms show transcription factors. Edges are indicated by activating or inhibiting arrows based on the sign of correlation between modules. Hub genes are highlighted with red borders.

**Figure 4:**
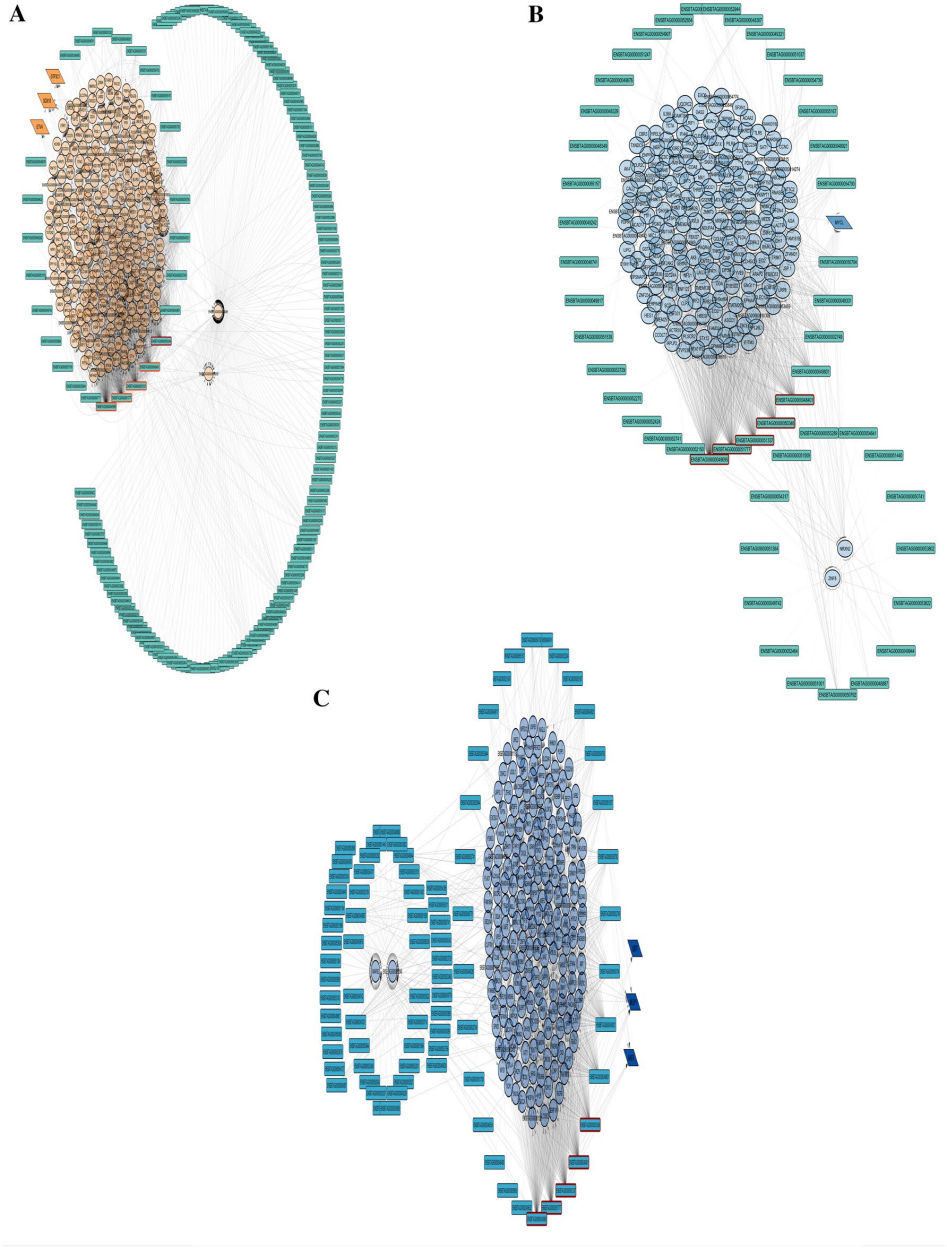
Regulatory network between turquoise lnc-module and (**A**) tan, (**B**) cyan and (**C**) midnight blue m-modules. lncRNAs are represented with rectangle shapes. In m-modules, ellipses show regular proteins and parallelograms show transcription factors. Edges are indicated by activating or inhibiting arrows based on the sign of correlation between modules. Hub genes are highlighted with red borders.

To better illustrate, key regulatory elements and hub genes in each relation between WGCNA-calculated co-expressed mi, lnc, and mRNAs expression values, visualized by Cytoscape. The architecture of the networks of each relation in Table 1 has shown in Figs. 3 and 4. The network constructed for the blue module of miRNA and turquoise lncRNA has been illustrated in Fig. 3a. Due to having a small number of genes, other relations of miRNA modules with lncRNA did not form a network. All edges are shown by activatory or inhibitory arrows based on the sign of the correlation between modules (inhibitory arrow for negative correlation and activatory arrow for positive correlation).

**Figure 5:**
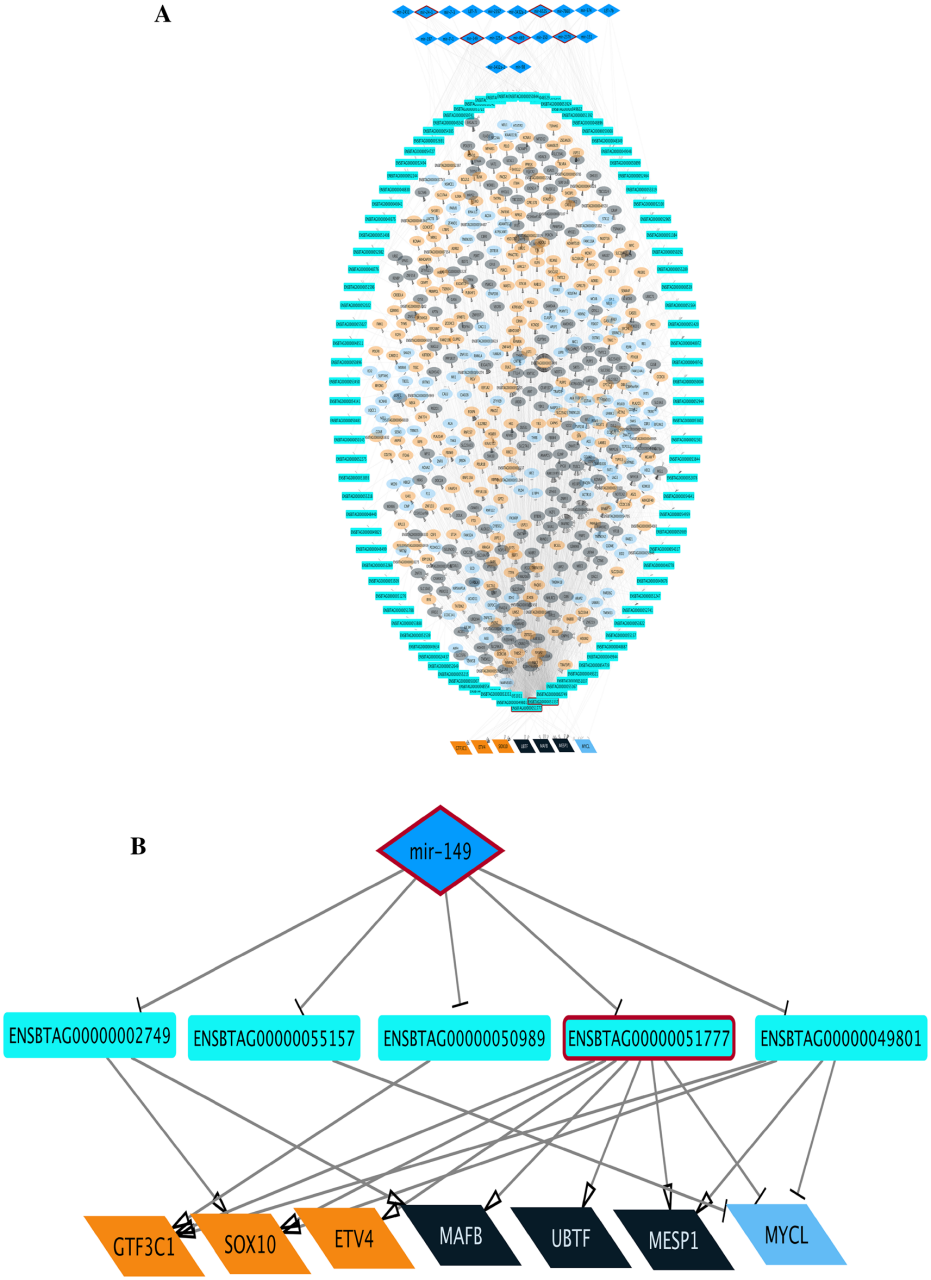
(**A**) Integrated lncRNA-miRNA-mRNA regulatory network of blue mi-modules, turquoise lnc-modules and tan, cyan and midnight blue m-modules. (**B**) Regulatory sub-network of the integrated lncRNA-miRNA-mRNA network. miRNAs are represented by diamonds, lncRNAs by rectangles, in m-modules ellipses show regular proteins and parallelograms show transcription factors. Edges are indicated by activating or inhibiting arrows based on the sign of correlation between modules. Nodes with red edges represent hub genes in the original networks.

The five top genes of each module (hub genes) detected by MCC topological analysis of CytoHubba have been highlighted in each network. Integration of all networks helps us to find miRNA–lncRNA–mRNA sub-networks to highlight specific molecular functions and mechanisms related to mastitis disease (Fig. 5). The brief statistic and hubs of each network were prepared in Supplementary Table S16 online. The name, biological kind, and module name of nodes and details of network statistics related to each network and sub-networks are presented in Supplementary Table S17 online.

## Discussion

Bovine mastitis is an inflammatory disease with clinical and subclinical symptoms, caused by a variety of infectious agents, controlled by a complex network of biological substances, resulting in significant economic losses due to negative effects on animal welfare, productive and reproductive performance, poor milk quality, increased workload, early culling and high treatment costs. Although transcriptional analysis of the bovine mammary gland in response to intramammary infection by *Str. uberis* reveals many immune-related genes and pathways, the regulatory elements, such as miRNAs, lncRNAs and TFs, involved in the expression of these genes are not yet fully understood. A growing number of reports suggest a significant utility of miRNAs, lncRNAs, and TFs as biomarkers of pathogenic conditions, modulators of drug resistance, and/or drugs for medical intervention.

Here, co-expression analysis and the integrated regulatory network approach were used to better understand the functional networks/pathways contributing to mastitis with *Str. uberis* infection. Through this research, we generated a list of miRNAs, lncRNAs and TFs that play critical roles in the response of mammary epithelial cells to Str. uberis infection. Functional analysis of intramodular hub genes detected by the WGCNA algorithm for semi- and non-preserved modules mostly enriched terms related to immune response, inflammation, cytokine and chemokine signalling, acute phase proteins, proteolysis and apoptosis. All networks constructed between the module relationships shown in Figs. 3 and 4. A total of, 18 miRNAs including mir-149, mir-6525, mir-669, mir-2376, mir-24-1, mir-10162, mir-12027, mir-2300a, mir-6521, mir-1248-1, mir-1248-2, mir-615, mir-133a-1, mir-2328-3p, mir-2328-5p, mir-2400, mir-29b-1, and mir-378-1, five lncRNAs including ENSBTAG00000048401, ENSBTAG00000049095, ENSBTAG00000050346, ENSBTAG00000051337, and ENSBTAG00000051777, and also seven TFs including SOX10, GTF3C1, ETV4, MYCL, MESP1, UBTF, and MAFB were identified as key regulators in mastitis caused by *Str. uberis* (Supplementary file S16).

Between them, mir-6525 and mir-669 were reported in a review on the role of non-coding RNAs in bovine mastitis diseases as dysregulated miRNAs during mastitis infection by Staphylococcus aureus and Streptococcus agalactiae, respectively^18^. Mir-149, as shown in our integrated network (Fig. 5a) and as a key regulator in the miRNA-lncRNA-mRNA sub-network (Fig. 5b), needs more attention.

It has been reported that suppression of bta-mir-149 promotes cell proliferation, migration and invasion, while inhibiting cell apoptosis^58^ at both transcriptional and post-transcriptional levels^59^ and may act as an oncogene (oncomiR) and tumour suppressor^60^. Bta-mir-149 has also been shown to inhibit pro-inflammatory cytokine production and its down-regulation correlates with increased expression of pro-inflammatory cytokines such as TNFα, IL1β and IL6^61^.

Based on the differential expression analysis results achieved from the original paper of our sequencing datasets^21^, bta-mir-149 was significantly down-regulated (−4 fold change) in mastitis samples compaired to control samples.

In Fig. 5a, mir-149 was shown to negatively regulate 40 lncRNAs and indirectly regulate 7 TFs and 572 protein-coding genes. We illustrated a new sub-network to focus more on TFs as key regulators seen in these m-modules (Fig. 5b). As shown in Fiure 5b ENSBTAG00000049801, ENSBTAG00000051777, and ENSBTAG00000055157 negatively regulate MYCL. Lung-derived MYC (MYCL) is a member of the myelocytomatosis oncogene (MYC) family of transcription factors, which recent evidence suggests plays an essential role in the regulation of cell growth, cell cycle, cell metabolism and cell death^62^. The role of MYC in immunosuppression and a significant opportunity in combining MYC inhibitors with immunotherapies has recently been reported^63^. MYCL has been found to be amplified or overexpressed in many tumour types^64^.

Based on our results down-regulated expression of bta-miR-149 indirectly inhibits MYCL transcription factor functions by suppressing its negative effects on ENSBTAG00000049801, ENSBTAG00000051777, and ENSBTAG00000055157 lncRNAs.

Furthermore, Fig. 5a has shown that bta-mir-149 indirectly promotes the expression of the transcription factors ETV4, GTF3C1, MAFB, MESP1, SOX10 and UBTF. These TFs are stimulated by the lncRNAs ENSBTAG00000055157, ENSBTAG00000051777, ENSBTAG00000050989, ENSBTAG00000049801 and ENSBTAG00000002749. The down-regulated expression of bta-mir-149 by suppressing its negative regulation on these lncRNAs promotes the action of downstream TFs. To explain the role of these TFs, ETV4 is involved in proliferation and induction of differentiation-associated genes^65^ and promotes breast cancer cells^66^, SOX10 has a positive regulation on epithelial cell proliferation^67^, GTF3C1 has been introduced as an immune-related marker for breast cancer^68^ by modulating cell proliferation, invasion, adhesion, angiogenesis and survival^69^, MAFB regulates dendritic cell maturation, the master regulator of the immune response^70^, is an inducer of monocytic differentiation^71^, and macrophage development^72^. Mesoderm posterior 1 (MESP1), which belongs to the family of basic helix-loop-helix transcription factors and is a master regulator of mesendoderm development, has also been shown to play a critical role in proliferation as a cancer oncogene gene^73^. The last TF, Upstream Binding Transcription Factor (UBTF), is known for transcriptional and chromatin modulation and has been reported to act as a transcriptional repressor of viral gene expression^74^. Regarding the related roles of these transcription factors, they can be regulated by the common pathways to manage the expression genes of bovine mastitis.Mir-615, detected as a hub in the network Fig. 3c constructed by the assigned royalblue mi-module and the green semi-preserved m-module, is another interesting miRNA in our results that needs more focus. A bovine miR-615 sequence obtained from miRbase (release Mar. 2023) is 92 nucleotides long and represents a highly conserved sequence^75^.

Research has shown conflicting results on the key role of Mir-615 as a tumour suppressor with an inhibitory role in cell proliferation, migration, and invasion^76,77^ or as a tumour promoter, inhibiting apoptosis and thereby contributing to tumour growth, proliferation, invasion and migration^78,79^. Apoptosis, as a highly regulated process of cell death required for the development and homeostasis of multicellular organisms, is a key feature of mammary gland development and function and is critical for the removal of milk-secreting alveolar epithelial cells during lactation and post-lactational involution^80^. Programmed cell death often accompanies the death of the infectious agent and may promote efficient clearance of the pathogen. Activation or prevention of cell death may be a critical factor in the outcome of infection^81^. Conclusive and direct evidence for the involvement of apoptosis in *Str. uberis*-induced mastitis has not been provided. Based on our results, mir-615 with significantly 16-fold up-regulated in DE analysis of the original paper of our datasets^21^ may be a mediator of apoptosis for *Str. uberis* infection. Searched results in enrichment analysis output of mir-615’s targets showed positive regulation of the apoptotic process has been significantly enriched by targets. Taken together, these findings illustrated that up-regulation of mir-615 through negative regulation of downstream targets may have an inhibitory role in apoptosis and may be a reason to justify the previous finding that illustrated *Str. uberis* can persistently colonise the mammary gland without elevating somatic cell count^82^.

Another hub miRNA presented in Fig. 3d, miR-29b, affects the lactation activity of dairy cow mammary epithelial cells by DNA hypermethylation of the promoters of important lactation-related genes^83^. Previous studies have shown that miR-29b is repressed by the NF-κB pathway, a key modulator of the inflammatory response^84^. The association of miR-24-1 (hub in Fig. 3a) with the NF-κB pathway has been previously reported^85^. Previous studies have shown that miR-29b is repressed by the NF-κB pathway, a key modulator of the inflammatory response^84^. The association of miR-24-1 (hub in Fig. 3a) with the NF-κB pathway has been reported previously^85^.

Previous research has shown that miR-133a (hub in Fig. 3c) exacerbates inflammatory responses by targeting and inhibiting the expression of sirtuin-1^86^.

Other hub miRNAs (mir-10162, mir-12027, mir-2300a, mir-2328-3p, mir-2376, mir-6521) are new and not enough information was found about them.

Our results also identified genes with the highest association with upstream regulators, including ABAT (Fig. 3b), ENSBTAG00000050205 (Fig. 4a), NRXN2 (Fig. 4b) and ENSBTAG00000052846 (Fig. 4c), which have not been previously reported in mastitis but may play an important role in this disease based on the following literature review results. GABA, the major inhibitory neurotransmitter, is reported to be a potent immunomodulatory molecule that is metabolised by the action of the enzyme Aminoutyrate aminotransferase (ABTA). Aminobutyrate aminotransferase (ABTA) has been detected in macrophages, CD4+ T cells and peripheral human monocytes^87^. The new gene ENSBTAG00000050205, shown in Fig. 4a, showed 99.81% identity to LTBP2 using blastn in NCBI^88^. Latent transforming growth factor-beta (TGF-beta)-binding protein (LTBP) has been shown to play a key role in apoptosis^89^.

NRXN2 as a potential regulator of inflammatory pain^90^ shown in Fig. 4b. New gene ENSBTAG00000052846 with 99.3% identity to NT5C3A using blastn software^88^ presented in Fig. 4c. Previous research suggests that NT5C3A mediates feedback inhibition of proinflammatory cytokine production by acting epigenetically to block NF-κB signalling output^91^.

## Conclusion

To improve our understanding of systems biology, it is crucial to gain insight into the regulatory components, such as miRNAs, lncRNAs and TFs, that have the potential to influence the expression of immune genes in the mammary gland upon exposure to a particular pathogen. These findings may provide a promising avenue for improving the diagnosis and treatment strategies for mastitis diseases in the dairy industry. Our research using the advanced capabilities of WGCNA, such as module detection and preservation analysis, has identified some potential regulatory genes (miRNA, lncRNA and TFs). Interestingly, most of these genes identified as regulators with significant roles in immune response, inflammation and apoptosis are novel in the field of mastitis. However, further experimental work is needed to validate our findings and elucidate the importance of these networks in bovine mastitis.

### Supplementary Information

Supplementary Table S1.

Supplementary Table S2.

Supplementary Table S3.

Supplementary Table S4.

Supplementary Table S5.

Supplementary Table S6.

Supplementary Table S7.

Supplementary Table S8.

Supplementary Table S9.

Supplementary Table S10.

Supplementary Table S11.

Supplementary Table S12.

Supplementary Table S13.

Supplementary Table S14.

Supplementary Table S15.

Supplementary Table S16.

Supplementary Table S17.

Supplementary Figure S1.

## Data Availability

The datasets analyzed during the current study are available in the NCBI's Gene Expression Omnibus (GEO) under the accession number GSE51856 and GSE51858, https://www.ncbi.nlm.nih.gov/geo/query/acc.cgi?acc=GSE51856 and https://www.ncbi.nlm.nih.gov/geo/query/acc.cgi?acc=GSE51858 respectively.
