# Supplementary material for: Integrated co-expression analysis of regulatory elements (miRNA, lncRNA, and TFs) in bovine monocytes induced by Str. uberis
Source: Sci Rep. 2023 Sep 12;13:15076. doi: 10.1038/s41598-023-42067-4 (PMC10497586; doi:10.1038/s41598-023-42067-4)
Supplement: Supplementary file 18 — Supplementary Figure S1. [file 41598_2023_42067_MOESM18_ESM.pdf]

Integrated co-expression analysis of regulatory elements (miRNA, lncRNA, and TFs) in bovine monocytes induced by *Str. uberis*

Somayeh Sharifi[1], Abbas Pakdel[1], Mohammad Hossein Pakdel[2], Raana Tabashiri[3], Mohammad Reza Bakhtiarizadeh[4], Ahmad Tahmasebi[5]

[1] Department of Animal Sciences, College of Agriculture, Isfahan University of Technology, Isfahan 84156–83111, Iran
[2] Department of Plant Molecular Biotechnology, National Institute of Genetic Engineering and Biotechnology (NIGEB), Tehran, Iran
[3] Agricultural Biotechnology Department, Tarbiat Modares University, Tehran, Iran
[4] Department of Animal and Poultry Science, College of Aburaihan, University of Tehran, Tehran 3391653755, Iran
[5] Institute of Biotechnology, Shiraz University, Shiraz 71946-84334, Iran

Corresponding authors:
Abbas. Pakdel, Somayeh Sharifi
Department of Animal Science at the Isfahan University of Technology, Isfahan, Iran
Postal Code: 84 8415683111
Pakdel@iut.ac.ir, ss.sharifi2015@gmail.com

Mean-variance relationship provided by voom function in the limma package (R software version 4.1.0)

a. lnc-modules b. mi-modules c. m-modules

a

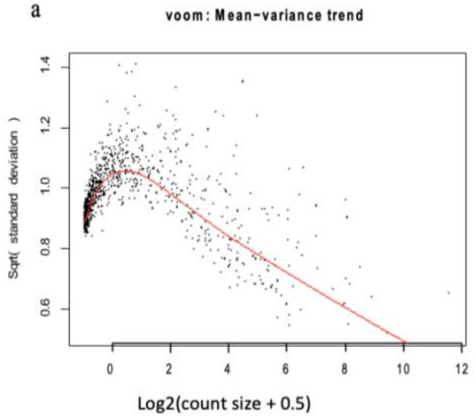

b

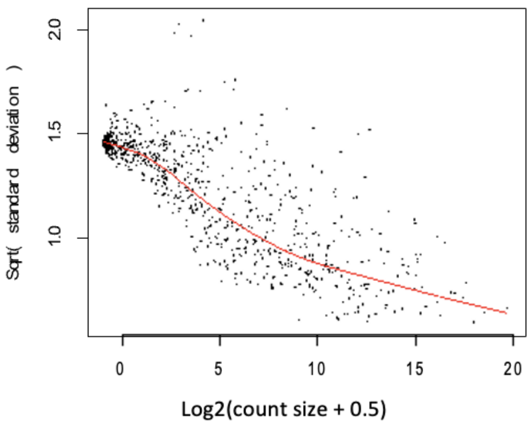

c

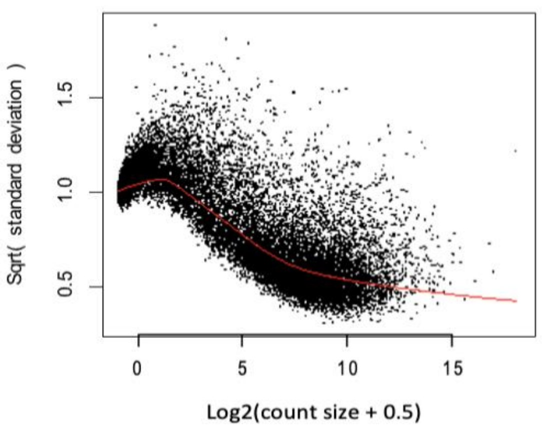

Analysis of network topology for a set of soft thresholding powers.

The left plot displays the scale of the free fit index (y-axis) as a function of the soft thresholding power (x-axis). The right plot shows the mean connectivity (degree, y-axis) as a function of the soft thresholding power (x-axis).

a. lnc-modules b. mi-modules c. m-modules

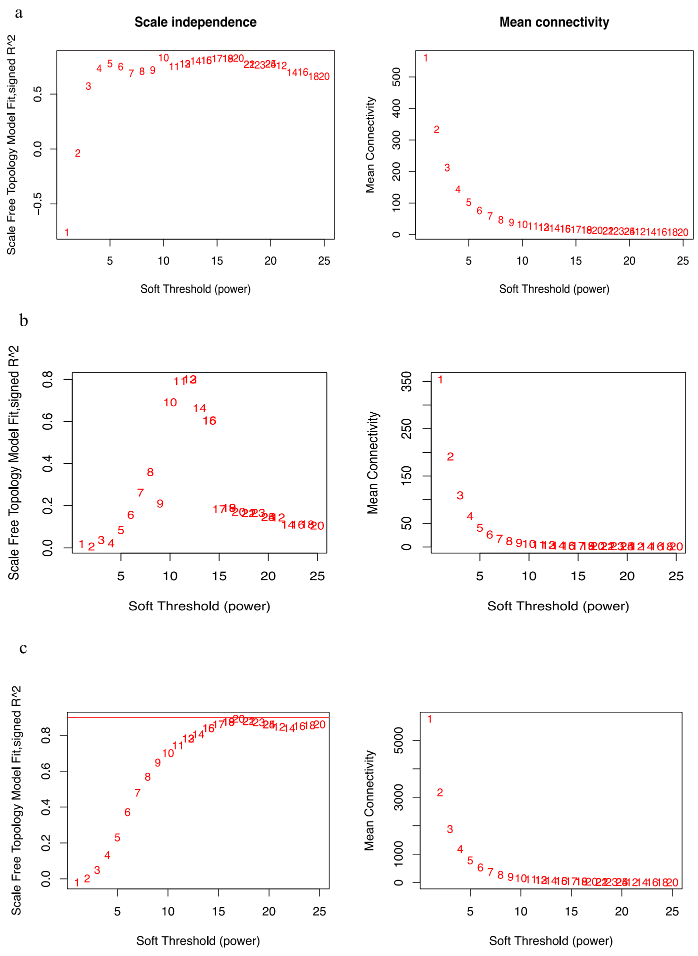

Clustring dendrogram of

a. lnc-modules b. mi-modules c. m-modules

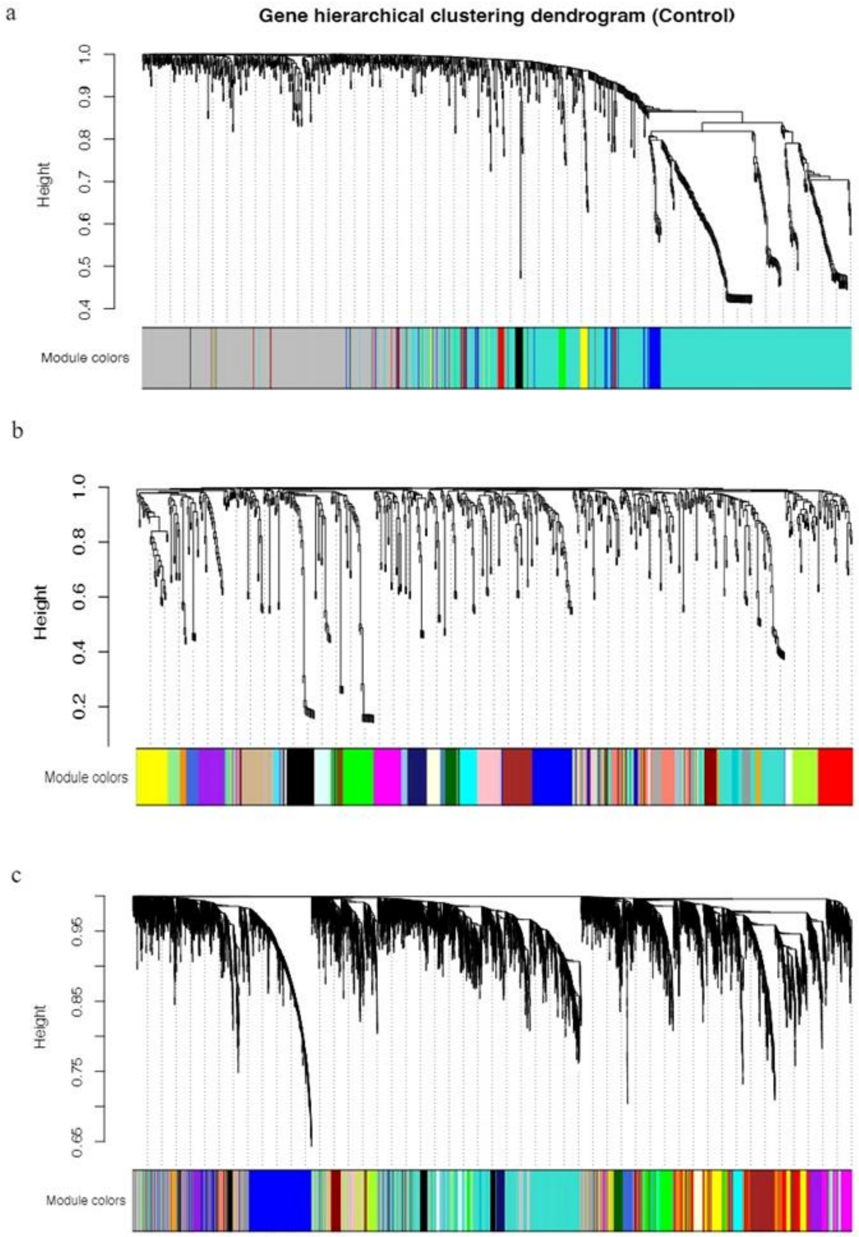

Correlation heatmaps of Mes

A. between mi-modules and m-modules, B: between lnc-modules and m-modules, and C. between lnc-modules and mi-modules

A

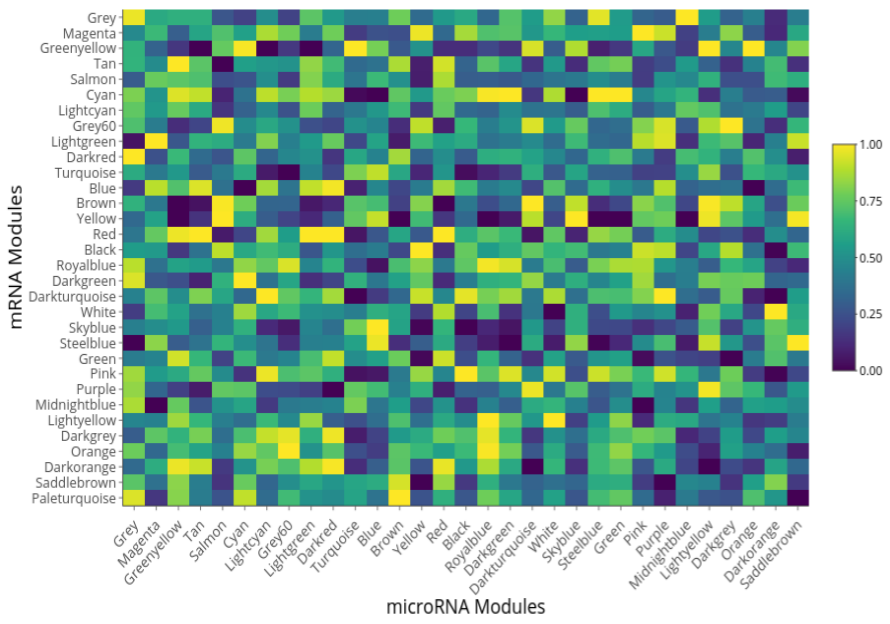

B

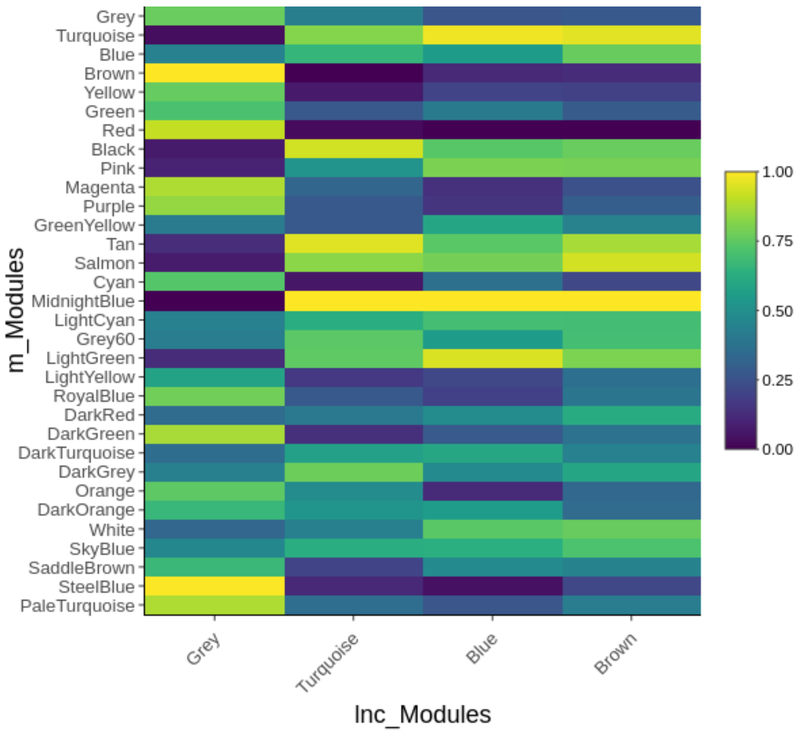

C

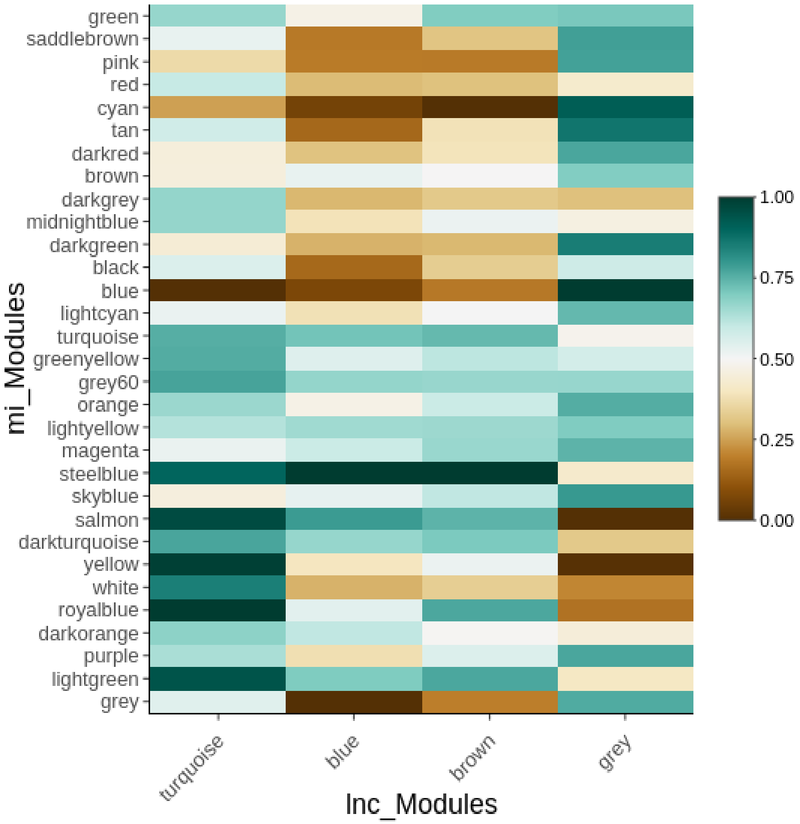

green
saddlebrown
pink
red
cyan
tan
darkred
brown
darkgrey
midnightblue
darkgreen
black
blue
lightcyan
turquoise
greenyellow
grey60
orange
lightyellow
magenta
steelblue
skyblue
salmon
darkturquoise
yellow
white
royalblue
darkorange
purple
lightgreen
grey
mi_Modules
turquoise
blue
brown
grey
lnc_Modules
1.00
0.75
0.50
0.25
0.00
